# Comparative Anti-Infectious Bronchitis Virus (IBV) Activity of (-)-Pinene: Effect on Nucleocapsid (N) Protein

**DOI:** 10.3390/molecules16021044

**Published:** 2011-01-25

**Authors:** Zhiwei Yang, Nan Wu, Yuangang Zu, Yujie Fu

**Affiliations:** 1 Key Laboratory of Forest Plant Ecology, Ministry of Education, Northeast Forestry University, Harbin 150040, China; 2 Engineering Research Center of Forest Bio-preparation, Ministry of Education, Northeast Forestry University, Harbin 150040, China; E-Mails: yzws-123@163.com (Z.Y.); bao_doubao@yahoo.com.cn (N.W.); fuyujie1967@yahoo.com.cn (Y.J.F.)

**Keywords:** (-)-pinene, anti-IBV activity, MTT, docking, active site

## Abstract

In the present study, anti-IBV (infectious bronchitis virus) activities of (-)-pinenes were studied by MTT assay, as well as docking and molecular dynamic (MD) simulations. The CC_50_values of (-)-α-pinene and (-)-β-pinene were above 10 mM. And the maximum noncytotoxic concentrations (TD_0_) of (-)-α-pinene and (-)-β-pinene were determined as 7.88 ± 0.06 and 6.09 ± 0.31 mM, respectively. The two compounds were found to inhibit IBV with an IC_50_ of 0.98 ± 0.25 and 1.32 ± 0.11 mM. The MTT assay showed that the inhibitions of (-)-pinenes against IBV appear to occur moderately before entering the cell but are much stronger occur after penetration of the virus into the cell. Molecular simulations indicated that (-)-α-pinene and (-)-β-pinene specifically interact with the active site which is located at the N terminus of phosphorylated nucleocapsid (N) protein, with the former being more potent than the latter. The binding energies of them are −36.83 and −35.59 kcal mol^−1^, respectively. Results presented here may suggest that (-)-α-pinene and (-)-β-pinene possess anti-IBV properties, and therefore are a potential source of anti-IBV ingredients for the pharmaceutical industry.

## 1. Introduction

Infectious bronchitis virus (IBV), which belongs to the family Coronaviridae, continues to be one of the most economically important pathogens in the poultry industry. Coronaviruses, which are enveloped viruses with positive sense, 5Vcapped and 3Vpolyadenylated RNA genomes, range from 27.6 to 32 kb [1]. Two thirds of the coronavirus genome encodes the replicase activity, including a viral RNA-dependent RNA polymerase (RdRp), helicase, and viral proteinases. The remaining one third of the genome encodes the structural proteins and small group-specific ORFs [1]. Recently, there have been occasional outbreaks of infectious bronchitis (IB) and it remains one of the most important poultry diseases in many countries of the world [2,3,4]. Consequently, study and exploiture an effective anti-IBV medicine have significant value and broad interest. 

IBV has four essential structural proteins: the three membrane proteins, the spike (S), integral membrane (M), and small envelope (E) proteins, and a phosphorylated, nucleocapsid (N) protein. The S protein interacts with cellular receptors and induces cell and viral membrane fusion [5]. The E and M proteins are localized in ER-Golgi intermediate compartment and considered to play critical roles in viral budding [6,7]. N proteins, which interact with viral genomic RNA, forming ribonucleocapsid (RNP) complexes, have been associated with replication and transcription [8,9,10]. It is a highly immunogenic phosphoprotein also implicated in viral genome replication and in modulating cell signaling pathways. The N-terminal of N-protein (NTD) serves as a functional unit critical for the specific interaction with RNA, where exhibits a U-shaped structure, with two arms rich in basic residues [11]. Additionally, the key functional residues of NTD are highly conserved among coronaviruses between different antigenic groups [12,13,14]. The N protein has been a major protein target in the exploration of anti-IBV medicine [11,12,13,14,15,16]. 

**Figure 1:**
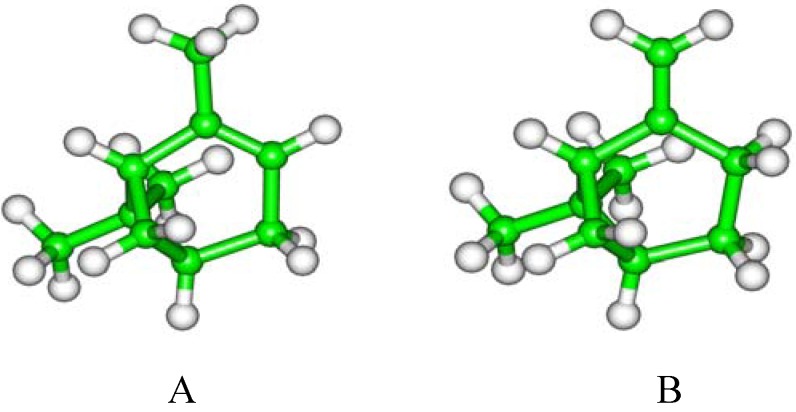
Structures of (-)-α-pinene (A) and (-)-β-pinene (B).

Aromatic and medicinal plants produce essential oils containing both hydrocarbons and oxygenated derivatives. In fact, essential oils have been widely used in traditional medicine. Among others, antibacterial, antifungal, immunomodulatory, antiinflammatory, and antirheumatic activities have been described [17,18,19]. (-)-Pinenes (α- and β-) (Figure 1) are major components of turpentine, the byproducts of the pulp making industry. Recent clinical research has shown that they present antimicrobial activities [20,21]. 

To the best of our knowledge, the anti-IBV activities of (-)-pinenes (α- and β-) have not been evaluated yet. Therefore, the aim of the present study was to evaluate the anti-IBV activities of (-)-α-pinene and (-)-β-pinene by MTT assay. Furthermore, explicitly solvated flexible docking and molecular dynamic (MD) methods were applied to investigate the inhibitory mechanisms of the two compounds with the IBV N protein. We anticipate that the insight into the understanding of binding mechanism will be of value in the rational design of IBV inhibitors. 

## 2. Results and Discussion

### 2.1. Effects of (-)-pinenes against IBV by MTT assay

Monolayer cultures of Vero cells were grown in 0.005–10 mM (-)-pinene-containing medium and after 72 h of incubation, cell viability was determined by MTT assay. The cytotoxicities of (-)-α-pinene and (-)-β-pinene on Vero cells were expressed as CC_50_ and TD_0_. As shown in Table 1, the CC_50_ values of (-)-α-pinene and (-)-β-pinene were all above 10 mM. And (-)-α-pinene showed higher maximum noncytotoxic concentration (TD_0_, 7.88 ± 0.06 mM) than (-)-β-pinene (6.09 ± 0.31 mM). These two values were both much higher than that of ribavirin (0.78 ± 0.15 mM) (*P* < 0.01). These results indicated compared with ribavirin, both (-)-α-pinene and (-)-β-pinene possessed lower cytotoxicity on Vero cells. Thus, we could infer that the antiviral effects of (-)-α-pinene and (-)-β-pinene were not due to any cytotoxicity.

**Table 1:** Anti-IBV activities of (-)-α-pinene and (-)-β-pinene compared with ribavirin.

Compound	CC_50_^a^ (mM)	TD_0_^b^ (mM)	IBV (Gray strain)	
			IC_50_^c^ (mM)	SI^d^
(-)-α-Pinene	>10.0	7.88 ± 0.06	0.98 ± 0.25	>10.20
(-)-β-Pinene	>10.0	6.09 ± 0.31	1.32 ± 0.11	>7.58
Ribavirin	>1.0	0.78 ± 0.15	0.118 ± 0.02	>8.47

Values in this table represent the mean values (±SD) of three independent experiments (*P* < 0.01). ^a, b^ Cytotoxic effect was determined by MTT assay. CC_50_ was the concentration that showed 50% cytotoxic effects in Vero cells. TD_0 _was the concentration that showed nontoxic maximum effects in Vero cells; ^c^ Antiviral activity was determined by MTT assay. IC_50_ was the concentration that inhibited 50% of IBV replication in Vero cells; ^d^ The selective index (SI) was calculated as CC_50_/IC_50_.

Moreover, the IC_50_ value of (-)-α-pinene (0.98 ± 0.25 mM) was somewhat lower than that of (-)-β-pinene (1.32 ± 0.11 mM). Based on the IC_50_ and CC_50_ values, the selectivity index (SI) was calculated as >10.20 and >7.58, respectively. It is reported that a SI of 4 or more should be appropriate for an antiviral agent [22]. This suggests that (-)-α-pinene and (-)-β-pinene may be judged to have significant anti-IBV activities.

### 2.2. Mode of anti-IBV activity by MTT assay

**Figure 2:**
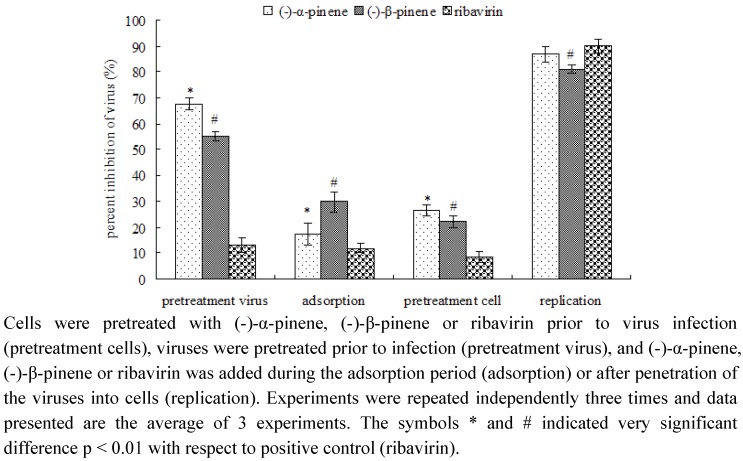
Antiviral effects of (-)-α-pinene (7.88 mM), (-)-β-pinene (6.09 mM) and ribavirin (0.78 mM) against IBV by incubation at different periods of time during infection.

To identify at which step replication might be inhibited, cells were infected with IBV after preincubation of the cells with ribavirin as positive control or pinenes, pretreatment of the virus with ribavirin or pinenes prior to infection, addition of the synthetic antiviral drug or pinenes during adsorption or after adsorption during the intracellular replication period. In all experiments cells infected with untreated virus were used as control. The percent reduction was calculated relative to the amount of virus produced in the absence of the drug. As shown in Figure 2, ribavirin showed the maximum antiviral activity when added at a concentration of 0.78 ± 0.15 mM during the replication period with inhibition of the viral replication of 90.18 ± 2.80% for IBV. As is commonly known, ribavirin interferes with RNA metabolism required for viral replication [23]. However, no significant effect was detected when ribavirin was used for pretreatment of cells or viruses or when ribavirin was only added during the adsorption phase. Unlike ribavirin, (-)-α-pinene showed the maximum noncytotoxic antiviral activity when added at a concentration of 7.88 ± 0.06 mM during the replication period with inhibition of the viral replication of 86.98 ± 3.04% for IBV, whereas, it also showed inhibition of 67.64 ± 2.31% in the pretreatment virus phase. Similarly to (-)-α-pinene, (-)-β-pinene also showed significant anti-IBV inhibition during the replication period and pretreatment virus phase, with the inhibition of 81.02 ± 1.48% and 55.41 ± 1.64%. These results suggested that the inhibitions of (-)-pinenes against IBV appear to moderately occur before entering the cell but much stronger occur after penetration of the virus into the cell. Additionally, biochemical studies indicated that the bioactivity of N protein is an important target for the replication of IBV virus [11,13,16]. Hence, we infer that N protein may be suppressed by (-)-pinenes.

### 2.3. NTD inhibitory activities of (-)-pinenes

In conclusion, (-)-α-pinene and (-)-β-pinene both possess anti-IBV properties by hindering the binding process between RNA and IBV N-protein. Our results support for the potential use of (-)-α-pinene and (-)-β-pinene in the treatment of IBV infectious disease. Further studies on the anti-IBV mechanism are needed to support this point of view. Therefore, explicit solvent docking and molecular dynamics (MD) simulations were used to explore the inhibiting mechanisms of (-)-α-pinene and (-)-β-pinene with NTD and to try to elucidate the activity differences.

**Figure 3:**
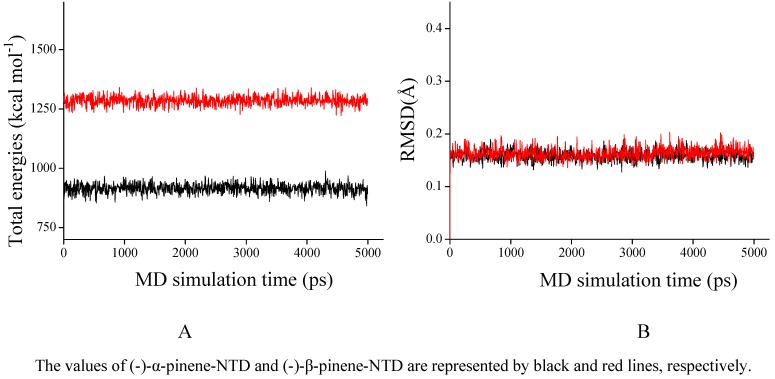
The time-evolution total energies (A) and backbone-atom root mean square deviations (RMSD, B) for the docked complexes during the MD simulations.

**Figure 4:**
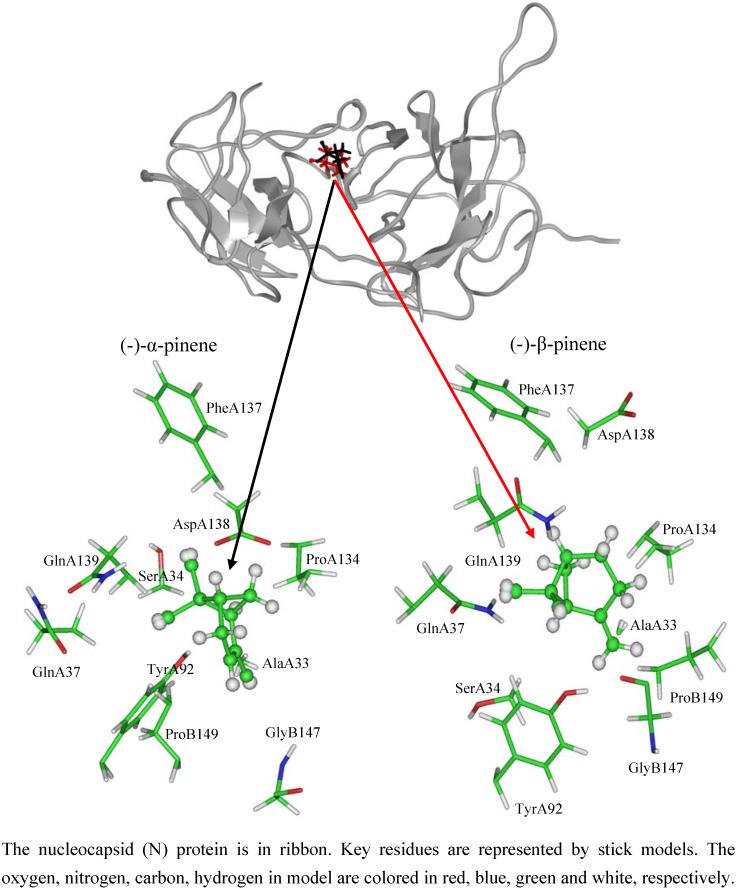
The modeled structures of (-)-α-pinene (black) and (-)-β-pinene (red) bound to the nucleocapsid (N) protein.

As shown in Figure 3, total energies and backbone-atom RMSDs indicated that the two docked complexes reached equilibrium after about 1,000 ps and remained rather stable afterwards. Accordingly, the geometric and energetic analyses were performed on the average structures of 1,000~5,000 ps MD trajectories. The superposed structures in Figure 4 showed that (-)-α-pinene and (-)-β-pinene occupy the proximity space at the RNA binding site of NTD, which is mapped to the loop region on the top of the β-sheet within the protein [11,14]. However, their binding modes differed from each other.

The interaction energy (*E_inter_*) of (-)-α-pinene with NTD was calculated to be -36.83 kcal mol^-1^. Van der Waals interactions rather than electrostatic interactions played a dominant role for the binding process, contributing to almost 86%. As Figure 4 shows, the cyclohexene ring of (-)-α-pinene was sandwiched between residues TyrA92 and ProA134 and exerted strong interactions with them. The values (*E_sum_*) amounted to −6.32 and −2.65 kcal mol^−1^, respectively (Table 2). Besides, (-)-α-pinene had strong van der Waals interactions (*E_vdW_*) with residues SerA34, GlnA37, PheA137, AspA138, GlnA139, GlyB147 and ProB149, especially residues PheA137 and ProB149, where the values were equaling −2.66 and −2.30 kcal mol^−1^, respectively (Figure 4 and Table 2). As these fully or partially conserved residues are key for the RNA bindings [11,14,16], it commendably support a viewpoint that (-)-α-pinene hindered the binding of RNA with NTD, which is in good agreement with the inhibition occurred strongly after penetration of the virus into the host cells.

Similarly, the binding of (-)-β-pinene was mainly stabilized by van der Waals interactions. The interaction energy (*E_inter_*) of (-)-β-pinene with NTD was slightly reduced to −35.59 kcal mol^−1^, with van der Waals energy (*E_vdW_*) owning 98% of it. As shown in Figure 4 and Table 2, the strong van der Waals effects were also observed between cyclohexane ring of (-)-β-pinene and the residues TyrA92 and ProA134 (Figure 4).

**Table 2:** The vdW, electrostatic and sum interaction energies (E*_vdW_*, E*_ele_* and E*_sum_*) involving (-)-α-pinene and (-)-β-pinene with the active-site residues of nucleocapsid (N) protein *^a^.*

	(-)-α-pinene			(-)-β-pinene		
Residue	E*_vdW_*	E*_ele_*	E*_sum_*	E*_vdW_*	E*_ele_*	E*_sum_*
AlaA33	—	—	—	−1.81	−0.36	−2.17
SerA34	−1.61	−0.18	−1.79	−3.03	−0.10	−3.13
PheA36	—	—	—	−1.07	−0.50	−1.57
GlnA37	−1.57	0.07	−1.50	−2.22	−0.11	−2.33
TyrA92	−3.86	−2.46	−6.32	−3.45	−0.14	−3.59
ProA134	−2.53	−0.12	−2.65	−2.96	−0.42	−3.38
PheA137	−2.66	0.08	−2.58	−2.00	−0.23	−2.23
AspA138	−1.43	−0.94	−2.37	−2.35	−0.31	−2.66
GlnA139	−2.21	−0.43	−2.64	−2.42	−0.23	−2.65
TyrA140	−1.66	0.08	−1.58	—	—	—
AspB146	−1.00	−0.89	−1.89	—	—	—
GlyB147	−0.77	−0.23	−1.00	−2.47	0.01	−2.46
GlyB148	—	—	—	−1.58	0.55	−1.03
ProB149	−2.30	−0.02	−2.32	−0.63	−0.09	−0.72
TrpB155	−1.85	−0.08	−1.93	−1.57	−0.04	−1.61

*^a^* Energy units in kcal mol^−1^.

The van der Waals contributions (*E_vdW_*) of the two residues were calculated to be −3.45 and −2.96 kcal mol^−1^, respectively (Table 2). The maximal binding difference between (-)-α-pinene-NTD and (-)-β-pinene-NTD is in that the methylene group (=CH_2_) of (-)-β-pinene was oriented towards residue AlaA33. As a result of this situation, the van der Waals contributions (E*_vdW_*) of residues PheA137 and ProB149 sharply reduced to −2.00 and −0.63 kcal mol^−1^, respectively. It indicated that the binding of RNA may interfer with (-)-β-pinene and this effect is lower than that of (-)-α-pinene, consistent with the above experimental data.

Taken together, it is likely that (-)-α-pinene and (-)-β-pinene exert their anti-IBV activities through the inhibition of binding process between RNA and IBV N protein, with the former having the higher bioactivity. Therefore, (-)-α-pinene and (-)-β-pinene should be potential lead compounds in the developing the anti-IBV agents. Further studies on the anti-IBV drugs are urgently needed to support this point of view.

## 3. Experimental

### 3.1. Materials

(-)-α-Pinene, (-)-β-pinene and ribavirin were obtained from Sigma Chemical Co. (St. Louis, MO, USA) and was stored in glass vials with Teflon sealed caps at −20 ± 0.5 °C in the absence of light.

### 3.2. Cell cultures

Vero-E6 (African green monkey kidney cells) was purchased from Harbin Veterinary Research Institute (Harbin, P. R. China). The cells were grown in monolayer culture with Dulbecco’s modified Eagle’s medium (DMEM) supplemented with 10% fetal calf serum (FCS), 100 U/mL penicillin and 100 μg/mL streptomycin. The monolayers were removed from their plastic surfaces and serially passaged whenever they became confluent. Cells were plated out onto 96-well culture plates for cytotoxicity and anti-IBV assays, and propagated at 37 °C in an atmosphere of 5 % CO_2_.

### 3.3. Viruses

The IBV Gray strain was purchased from National Control Institute of Veterinatory Bioproducts and Pharmaceuticals (Beijing, P. R. China). Virus was routinely grown on Vero-E6 cells. IBV-Gray stock cultures were prepared from supernatants of infected cells and stored at −80 °C.

### 3.4. Cytotoxicity assay

The cellular toxicity of (-)-α-pinene and (-)-β-pinene on Vero-E6 cells was assessed by the MTT method [24]. Briefly, cells were seeded on a microtiter plate in the absence or presence of various concentrations (10 mM – 0.005 mM) of (-)-α-pinene and (-)-β-pinene for eight replicates and incubated at 37 °C in a humidied atmosphere of 5% CO_2_ for 72 h. The supernatants were discarded, washed with PBS twice and MTT reagent (5 mg/mL in PBS) was added to each well, after incubated at 37 °C for 4 h, remove the supernatants, then 200 μL DMSO was added and incubated at 37 °C for another 30 min. After that the plates were read on an ELISA reader (Thermo Molecular Devices Co., Union City, USA) at 570/630 nm. The mean OD of the cell control wells was assigned a value of 100%. The maximal non-toxic concentration (TD_0_) and 50% cytotoxic concentration (CC_50_) were calculated by linear regression analysis of the dose-response curves generated from the data.

### 3.5. Anti-IBV activity

Inhibition of virus replication was measured by MTT method [25]. Serial dilution of the treated virus was adsorbed to the cells for 1 h at 37 °C. The residual inoculum was discared and infected cells were added with DMEM containing 2% FCS. Each assay was performed in eight replicates. After incubation for 72 h at 37 °C, the cultures were measured by MTT method as described above. The concentration of (-)-α-pinene, (-)-β-pinene and ribavirin which inhibited virus numbers by 50% (IC_50_) was determined from dose-response curves.

### 3.6. Mode of anti-IBV activity

Cells and viruses were incubated with (-)-α-pinene or (-)-β-pinene at different stages during the viral infection cycle in order to determine the mode of antiviral action [24]. Cells were pretreated with (-)-α-pinene or (-)-β-pinene before viral infection, viruses were incubated with (-)-α-pinene or (-)-β-pinene before infection and cells and viruses were incubated together with (-)-α-pinene or (-)-β-pinene during adsorption or after penetration of the virus into the host cells. (-)-α-pinene or (-)-β-pinene was always used at the nontoxic concentration. Cell monolayers were pretreated with (-)-α-pinene or (-)-β-pinene prior to inoculation with virus by adding (-)-α-pinene or (-)-β-pinene to the culture medium and incubation for 1h at 37 °C. The compound was aspirated and cells were washed immediately before the IBV inoculum was added. For pretreatment virus, IBV were incubated in medium containing (-)-α-pinene or (-)-β-pinene for 1h at room temperature prior to infection of Vero-E6 cells. For analyzing the anti-IBV inhibition during the adsorption period, the same amount of IBV was mixed with the drug and added to the cells immediately. After 1h of adsorption at 37 °C, the inoculum was removed and DMEM supplemented with 2% FCS were added to the cells. The effect of (-)-α-pinene or (-)-β-pinene (or ribavirin) against IBV was also tested during the replication period by adding it after adsorption, as typical performed in anti-IBV susceptibility studies. Each assay was run in eight replicates. Ribavirin was used as a positive control.

### 3.7. Flexible docking and MD simulations

The protein receptor N-terminal domain of N-protein (PDB ID: 2GEC) [11] from the RCSB Protein Data Bank was taken without the crystal water molecules [14]. For convenience, it is named as NTD throughout this work. The geometries and partial atomic charges of (-)-α-pinene and (-)-β-pinene (Figure 1) were taken by applying the BFGS algorithm (Discover 3.0 module) [26], with the consistent-valence force-field (CVFF). The convergence criterion was set to 0.01 kcal mol^−1^ Å^−1^. Demonstrated by previous literatures [14,27,28,29], the explicitly solvated flexible docking and molecular dynamics (MD) simulations were performed by the general and popular protocols in the InsightII 2005 software packages [30] on Linux workstations. The MD trajectories were generated using a 1.0-fs time step for a total of 5,000 ps, saved at 5.0-ps intervals. The interaction energies of the compounds with NTD and the respective residues at the NTD active site were calculated by the Docking module [31], over the 1,000~5,000 ps MD trajectories. More calculated details are referred elsewhere [27,29]. 

### 3.8. Statistical analysis

All results are expressed as mean values ± standard deviations (SDs) (n = 3). The significance of difference was calculated by one-way analysis of variance, and values *p* < 0.01 were considered to be significant.

## 4. Conclusions

The current study demonstrated that the certain anti-IBV activity as well as the inhibition of binding process between RNA and IBV N protein of (-)-α-pinene and (-)-β-pinene. Further pharmacological investigations are necessary to provide evidence about the anti-IBV mechanism of these two compounds. 
